# Identification of genes associated with environmental persistence in *Campylobacter jejuni* and *Campylobacter coli* isolates from processing in a broiler abattoir

**DOI:** 10.1007/s11259-022-09981-w

**Published:** 2022-09-12

**Authors:** A. Carbonero, A. Maldonado-Iniesta, Y. Trujillo, J. Perea, M. Riofrío, I. Garcia-Bocanegra, C. Borge

**Affiliations:** 1 Department of Animal Health, University of Cordoba, Animal Health Building, Campus Universitario de Rabanales, 14014 Córdoba, Spain; 2 Department of Animal Production, University of Cordoba, Production Animal Building, Campus Universitario de Rabanales, 14014 Córdoba, Spain; 3 Andalusian Health Service, Health Center Polígono del Guadalquivir, 14013 Cordoba, Spain

**Keywords:** *Campylobacter coli*, *C. jejuni*, Environmental persistence, Genes, Poultry

## Abstract

The aim of this study was to determine the prevalence of the *htr*A, *htr*B and *ppk*1 genes -all of which are related to environmental persistence- in *C. jejuni* and *C. coli* isolates obtained from abattoir samples at the arrival of broilers (initial stage) and in meat products after processing (final stage). A total of 119 DNA extracts (55 *C. jejuni* and 64 *C. coli*) were included in the study*.* Identification of genes was performed by conventional PCR (one for each gene). The overall prevalence was 40.3%, 93.3% and 68.9% for the *htr*A, *htr*B and *ppk*1 genes, respectively. Statistically significant differences were found (*p* < 0.05) between prevalence of *C. jejuni* and *C. coli* for all three genes. In *C. coli* the prevalence was significantly higher for the *htr*A (*p* = 0.007) and *htr*B (*p* = 0.015) genes, while *ppk*1 gene prevalence was significantly higher in *C. jejuni* (*p* < 0.001). In addition, statistically significant increase in the frequency of *htr*A (*p* = 0.007) and *htr*B (*p* = 0.013) genes in the final product compared to broilers on arrival at the abattoir was observed in *C. jejuni*, but not in *C. coli.* These results suggest that *htr*A and *htr*B genes are involved in environmental persistence of *Campylobacter jejuni.*

## Introduction

In 2018, 246,571 cases of human campylobacteriosis were confirmed in the European Union. It is the most reported zoonosis since 2005 (EFSA and OCDE 2019). The species most frequently implicated in campylobacteriosis outbreaks are *Campylobacter jejuni* and *Campylobacter coli* (around 90% and 10% of the cases, respectively) (Wagenaar et al. 2013; Mossong et al. 2016). There are many domestic and wild reservoirs of *Campylobacter* species, but poultry are considered the main ones (Wagenaar et al. 2013; Josefsen et al. 2015). Meat contamination by *Campylobacter* spp. usually increases in broiler abattoirs along the processing (Ghafir et al. 2007; Torralbo et al. 2015). The main reason is the cross contamination that happens more frequently during scalding, evisceration and quartering stages (Torralbo et al. 2015; Seliwiorstow et al. 2016).

Bacteria belonging to *Campylobacter* genus are markedly labile in the external environment. Several conditions, such as the presence of oxygen, variations in pH or temperature, dryness and chemical products, can produce cell damage (Silva et al. 2011; Hofreuter 2014). However, a higher resistance of *C. coli* when compared with *C. jejuni* during processing at abattoir has been reported (Ma et al. 2014; Oh et al. 2015; Vinueza-Burgos et al. 2017).

The presence and the expression of some genes, such as *htr*B, *htr*A and *ppk*1, have been associated with a greater resistance to adverse environmental conditions. Previous studies about *Campylobacter jejuni* attributed different functions to *htr*A gene, such as greater tolerance to oxygen, resistance to thermal shock or invasion and colonization functions (Hanning et al. 2010; Baek et al. 2011; Boehm et al. 2015). Regarding *htr*B gene, it has an important role in promoting *Campylobacter* resistance against changes in pH, thermal stress, aerobic media and osmotic stress (Phongsisay et al. 2007; Bronowski et al. 2014). Finally, the *ppk*1 gene participates in the expression of poly-P, an inorganic polyphosphate responsible for the responses of the bacterium to conditions of lack of nutrients and osmotic stress, as well as the formation of biofilms (Candon et al. 2007; Drozd et al. 2014).

The objective of this study was to confirm the next hypothesis: if *htr*A, *htr*B and *ppk*1 genes are involved in environmental persistence of *C. jejuni* and *C. coli*, isolates having these genes will be selected under adverse conditions, such as those present during the processing at abattoir. Consequently, prevalence of isolates with these genes after processing would be significantly higher compared with prevalence before processing.

## Material and methods

DNA isolates from a previous study (Torralbo et al. 2015) performed by our group were used. Sampling of the study was carried out along 2012 in a slaughterhouse located near Malaga (Spain). In this abattoir about 60,000 chickens are slaughtered and quartered each day. It is divided in six areas/stages: loading dock, scalding, evisceration, classified, quartering and final meat product/packing. Only DNA extracts from samples obtained in the first (cloacae from broilers in the loading dock) and the last stage (quartered carcasses surfaces (breast, wing, leg and back)) were used in this study.

After sampling, swabs were cultured in *Campylobacter* Free Blood Agar (Oxoid®) with CCDA supplement (Oxoid®). Bulk colonies for each isolate in blood agar plate were taken with a loopful of 10 µl to carry out DNA extraction according to the method described by Sambrook and Russell (2006). Phenotypic characterization using catalase and oxidase test, Gram stain for shape observation and mobility test under dark light microscopy, were performed in a first stage. Compatible *Campylobacter* isolates were confirmed by Multiplex PCR (Torralbo et al. 2015). Fifty-five from 86 *C. jejuni* isolates and 64 from 80 *C. coli* isolates were randomly selected for this study. Forty-four isolates (31 *C. jejuni* and 13 *C. coli*) were obtained from the initial stage (cloacae from broilers in the loading dock) and 75 (24 *C. jejuni* and 51 *C. coli*) from the final stage (meat ready for retail). The difference in the number of isolates selected from the initial and final stage is due to the greater number of isolates obtained in the final stage in the previous study from which *Campylobacter* was isolated.

Conventional PCR was used to identify the genes related to environmental persistence *htr*A, *htr*B and *ppk*1. The strain of *C. coli* DSMZ 4689 was used as positive control. The PCR protocols described by Nierop Groot et al. (2014), Ghunaim et al. (2015), and Candon et al. (2007) were used to identify the *htr*A, *htr*B and *ppk*1 genes, respectively. Information about the molecular weight of the PCR products and sequences of primers is shown in the Table 1. The polymerase kit MyTaq Red Mix®, from Bioline, which includes the polymerase, the nucleotides, the magnesium chloride and the load buffer was used to perform the PCR.

**Table 1:** Primers used for detection of *htr*B, *htr*A and *ppk*1 genes

Gen	*Primers*	Pb	Reference
htrA	Fw (5’-AAA CTT TAG CCT AAG TCA ATC AAG-3’) Rv (5’-AAT TTA TAA CGA AAG GAA AAT CC-3’)	700	Nierop Groot et al. 2014
htrB	Fw (5’-CGC ACC CAA TTT GAC ATA GAA C-3’)	70	Ghunaim et al. 2015
	Rv (5’-TTT TTA GAG CGC TTA GCA TTT GTC T-3’)		
ppk1	Fw (5’-GCA AAT ATT TAC ACC AAG AAA AAG AAC-3’)	1550	Candon et al. 2007
	Rv (5’-ATC TGC ACT CGA TAT AAA ATA ATT TGG-3’)		

SPSS software (v. 22.0) was used to perform the statistical analyses. The initial, final and global prevalence of each gene was calculated for *C. jejuni* and *C. coli.* In addition, a Chi Squared test (χ^2^) was performed to establish the existence of significant differences between the prevalence of the genes in the initial and the final stage (p < 0.05). The same test was used to determine the existence of significant differences, at 95% confidence level, between the prevalence of *C. jejuni* and *C. coli*.

The procedures employed were ethically reviewed and approved by the bioethical committee of the University of Cordoba. The procedures employed in the submitted manuscript were performed only at laboratory level, in spaces approved by the biosecurity committee of the University of Cordoba.

## Results

The overall prevalence of the *htr*A, *htr*B and *ppk*1 genes was 40.3% (48/119), 93.3% (111/119) and 68.9% (82/119), respectively.

**Fig. 1:**
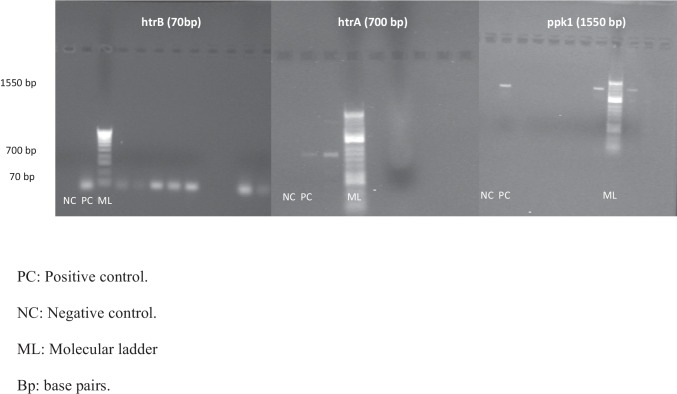
PCR results from *C. jejuni* DNA isolated from chicken simples from abbatoirs

**Fig. 2:**
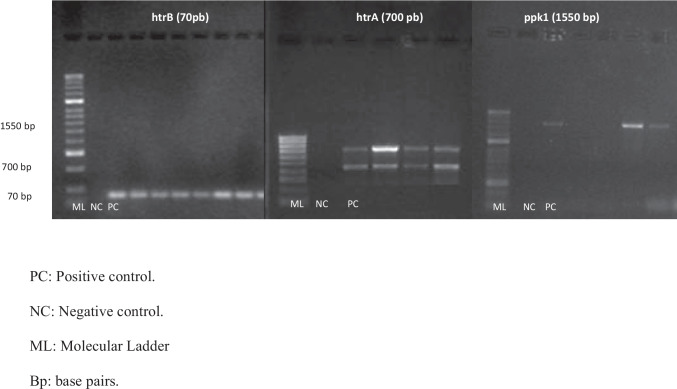
PCR results from *C. coli* DNA isolated from chicken simples from abattoirs

Table 2 shows the prevalence of the different genes in the initial and final stage for both *Campylobacter* species, *C. jejuni* and *C. coli*. Prevalence of *htr*A and *htr*B is significantly higher (p < 0.05) in the final stage when both species where included together. Significant differences are appreciated for *htr*A and *htr*B genes in *C. jejuni,* but not for *C. coli.* PCR examples for detection of *C. jejuni* and *C. coli* can be found in Figs. 1 and 2, respectively.

**Table 2:** Initial and final prevalence of *htr*B, *htr*A and *ppk*1 genes for *Campylobacter* spp., *C. jejuni* and *C. coli* and study of the statistical differences between both stages

	Gene	Initial product (Positive)	Final product (Positive)	Chi-squared^*^ (χ^2^)	*p*
*Campylobacter* spp.	*htr*A	9/44 (20.5%)	39/75 (52.0%)	11.47	0.001
	*htr*B	37/44 (84.1%)	74/75 (98.7%)	9.40	0.002
	*ppk*1	32/44 (72.7%)	50/75 (66.7%)	0.48	0.491
*C. jejuni*	*htr*A	4/31 (12.9%)	11/24 (45.8%)	7.40	0.007
	*htr*B	24/31 (77.4%)	24/24 (100%)	6.21	0.013
	*ppk*1	24/31 (77.4%)	23/24 (95.8%)	3.69	0.055
*C. coli*	*htr*A	5/13 (38.5%)	28/51 (54.9%)	1.12	0.290
	*htr*B	13/13 (100%)	50/51 (98%)	0.26	0.611
	*ppk*1	8/13 (61.5%)	27/51 (52.9%)	0.31	0.578

^*^Between initial and final prevalence

In Table 3 the prevalence of *htr*A, *htr*B and *ppk*1 genes in *C. jejuni* and *C. coli* is compared, showing that the prevalence of *htr*A and *htr*B genes is significantly higher in *C. coli* while prevalence of *ppk*1 gene is significantly higher in *C. jejuni.*

**Table 3:** Prevalence of *htr*B, *htr*A and *ppk*1 genes for *Campylobacter jejuni* and *C. coli* and study of the statistical differences between both species

	*C. jejuni* (Positive)	*C. coli* (Positive)	Chi-squared^*^ (χ^2^)	*p*
*htr*A	15/55 (27.3%)	33/64 (51.6%)	7.25	0.007
*htr*B	48/55 (87.3%)	63/64 (98.4%)	5.88	0.015
*ppk*1	47/55 (85.5%)	35/64 (54.7%)	13.07	< 0.001

^*^Between initial and final prevalence

## Discussion

In the present study we tried to support the hypothesis that *htr*A, *htr*B and *ppk*1 genes are involved in the environmental persistence of *Campylobacter* species. To the best of our knowledge, their role had only been evaluated by mean of laboratorial studies, but no previous field studies have been performed.

The main hypothesis in our study was that if these genes are involved in environmental persistence, their prevalence should increase since the strains having these genes would be positively selected during the stressful conditions existing along the processing at the abattoir. It only could be confirmed for *htr*A and *htr*B in *C. jejuni* at a confidence level of 95%*.* Differences in *ppk*1 for *C. jejuni* would be significant decreasing the signification level (p = 0.055).

Our results show that the three genes probably play a role in the environmental persistence of *C. jejuni,* but not in *C. coli* (Table 2). In contrast, a similar study elaborated by Hanning et al. (2010) with different genes related to the bacterial survival (*rac*R, *sob*B, *htr*A and *clp*A), no association with actual survival was observed.

The last part of our study consisted of the comparison of the prevalence of these three genes between *C. jejuni* and *C. coli* at the first and last stage in the slaughterhouse. A significant increase in the prevalence of *htr*A (from 27.3 to 51.6%) and *htr*B (from 87.3 to 98.4%) was observed in *C. coli* (Table 3)*.* The higher prevalence of these genes could explain the results reported by Torralbo et al. (2015), who described an increase in the relative prevalence of *C. coli* (compared with that one by *C. jejuni*) along the processing from the initial phase (41.5%) to the final stage (64.6%). There are also other studies describing a greater presence of *C. coli* at the end of processing at the slaughterhouse (Ma et al. 2014; Vinueza-Burgos et al. 2017).

On the other hand, the prevalence of *ppk*1 gene was significantly higher in *C. jejuni* (from 54.7 to 85.5%) (Table 3). This gene has been mainly related to the formation of biofilms, which has been extensively described in *C. jejuni* (Candon et al. 2007; Drozd et al. 2014), but scarcely for *C. coli.*

Resistance could be related not only to the presence of the gene but also with its level of expression. However, conventional PCR only provide information about the presence. Therefore, it would be convenient in the future to carry out novel studies using quantitative techniques (detection of DNA by qPCR or quantification of mRNA). It would be interesting also to continue the study including human isolations to evaluate if the presence of these genes is associated with an increase of human cases.

As conclusion, it has been observed that *htr*A, *htr*B and *ppk*1 genes are present in the isolates of *C. jejuni* and *C. coli* studied with medium to high prevalence. The prevalence of *htr*A and *htr*B significantly increased along the processing in *C. jejuni,* but not in *C. coli.* However, the prevalence of these two genes was significantly higher in *C. coli.* In contrast, the prevalence of *ppk*1 gene was significantly higher in *C. jejuni.*

## Data Availability

The datasets generated during and/or analysed during the current study are available from the corresponding author on reasonable request.
